# Cinacalcet may suppress kidney enlargement in hemodialysis patients with autosomal dominant polycystic kidney disease

**DOI:** 10.1038/s41598-021-89480-1

**Published:** 2021-05-11

**Authors:** Shinya Nakatani, Kozo Nishide, Senji Okuno, Eiji Ishimura, Daijiro Kabata, Fumiyuki Morioka, Yuri Machiba, Hideki Uedono, Akihiro Tsuda, Shigeichi Shoji, Masaaki Inaba, Katsuhito Mori, Tomoyuki Yamakawa, Masanori Emoto

**Affiliations:** 1 Department of Metabolism, Endocrinology and Molecular Medicine, Osaka City University Graduate School of Medicine, 1-4-3 Asahi-machi, Abeno-ku, Osaka, 545-8585 Japan; 2 Kidney Center, Shirasagi Hospital, Osaka, Japan; 3 Department of Nephrology, Meijibashi Hospital, Osaka, Japan; 4 Department of Medical Statistics, Medicine, Osaka City University Graduate School of Medicine, Osaka, Japan; 5 Department of Nephrology, Osaka City University Graduate School of Medicine, Osaka, Japan

**Keywords:** Nephrology, Urology

## Abstract

A massively enlarged kidney can impact quality of life of autosomal dominant polycystic kidney disease (ADPKD) patients. A recent in vitro study demonstrated that an allosteric modulator of the calcium sensing receptor decreases adenosine-3′,5′-cyclic monophosphate, an important factor for kidney enlargement in ADPKD. Therefore, the present study was performed to determine whether cinacalcet, a calcium sensing receptor agonist, suppresses kidney enlargement in hemodialysis patients with ADPKD. Alteration of total kidney volume together with clinical parameters was retrospectively examined in 12 hemodialysis patients with ADPKD treated at a single institution in Japan. In the non-cinacalcet group with longer hemodialysis duration (n = 5), total kidney volume had an annual increase of 4.19 ± 1.71% during an overall period of 877 ± 494 days. In contrast, the annual rate of increase in total kidney volume in the cinacalcet group (n = 7) was significantly suppressed after cinacalcet treatment, from 3.26 ± 2.87% during a period of 734 ± 352 days before the start of cinacalcet to − 4.71 ± 6.42% during 918 ± 524 days after initiation of treatment (*p* = 0.047). The present findings showed that cinacalcet could be a novel therapeutic tool for suppression of kidney enlargement in hemodialysis patients with ADPKD.

## Introduction

Autosomal dominant polycystic kidney disease (ADPKD) is the most common hereditary cause of chronic kidney disease (CKD) and the fourth leading cause of kidney failure in patients who underwent kidney replacement therapy (KRT) worldwide^1,2^, with approximately 50% of patients with ADPKD requiring KRT before the age of 60^3^. As kidney function deteriorates, total kidney volume (TKV) generally decreases in most patients with various types of CKD. In contrast, TKV in ADPKD patients usually continues to increase, even after initiation of dialysis therapy, because normal renal parenchyma is replaced by numerous cysts^4^. At the initiation of KRT, a cyst-filled kidney can weigh as much as 9–13 kg^5^, then as the cysts grow, ADPKD patients suffer from assorted uncomfortable complications, such as kidney pain, back pain, palpable kidneys, nephrolithiasis, urinary tract infection, gross hematuria, and gastrointestinal symptoms^6^. Of those, gastrointestinal symptoms, such as appetite loss and nausea, are clinically important, because an enlarged kidney associated with ADPKD has been reported to be a risk factor for malnutrition^7^, a strong predictor of mortality and morbidity in patients receiving dialysis^8,9^. Occasionally, kidneys that are massively enlarged require volume reduction intervention to relieve symptoms and improve quality of life^4^. Several methods, including needle aspiration and sclerosing of enlarged kidney cysts, fenestration of the cysts, a nephrectomy, and transcatheter arterial embolization (TAE), are available and have been found to be effective, though such intervention is generally accompanied by clinical complications, including bleeding, severe pain, and fever^4^. Thus, an effective safe therapy that suppresses progression of kidney enlargement in KRT patients with ADPKD needs to be developed.

Previously reported in vitro and in vivo studies have noted that calcium signaling dysregulation, represented by an elevated adenosine-3′,5′-cyclic monophosphate (cAMP) level, is a pivotal promoter of kidney-cyst cell proliferation and luminal fluid secretion^10–14^. In ADPKD patients, treatment with tolvaptan, a vasopressin V_2_ receptor antagonist, has been reported to suppress cAMP levels in the kidney, and also inhibit cell proliferation and kidney enlargement in a dose-dependent manner^15,16^. Furthermore, its efficacy to prevent enlargement of the kidneys and kidney function deterioration has been established by the results of two different pivotal clinical trials^17,18^. However, tolvaptan cannot be administrated to patients with kidney failure, because its efficacy and safety in such cases has not been proven. A previous in vivo study demonstrated that injection of parathyroid hormone (PTH) or long-acting PTH analog administration in mice led to an increase in cAMP levels in blood as well as in the kidneys^19^. Vitamin D receptor agonists (VDRA) could have the possibility to reduce increase in TKV. Although VDRAs have been available for patients with kidney failure for a long period, no reports regarding their efficacy to prevent enlargement of the kidneys in patients with ADPKD have been presented. Targeting somatostatin receptors with long-acting somatostatin analogues such as octreotide has also been shown to reduce cAMP accumulation and disease progression in experimental models of ADPKD^20^. However, that has yet to be shown in hemodialysis ADPKD patients. In consideration of the central role of cAMP in the pathogenesis of ADPKD progression, strategies to lower the level of cAMP in cystic tissues of ADPKD patients with kidney failure are necessary.

A recent in vitro study of human conditionally immortalized proximal tubular epithelial cells carrying the PKD1 mutation demonstrated that selective calcium sensing receptor (CaSR) activation by NPS-R568, a first-generation calcimimetic compound^21^, increased intracellular calcium and reduced cAMP levels^22^. The findings of the previous study^22^ suggested a potential therapeutic effect of cinacalcet to suppress kidney enlargement in ADPKD patients with hemodialysis. Hence, the present retrospective observational study was conducted to determine the effects of cinacalcet hydrochloride on kidney volume changes in hemodialysis ADPKD patients with secondary hyperparathyroidism.

## Results

### Clinical characteristics of hemodialysis patients with ADPKD

**Figure 1:**
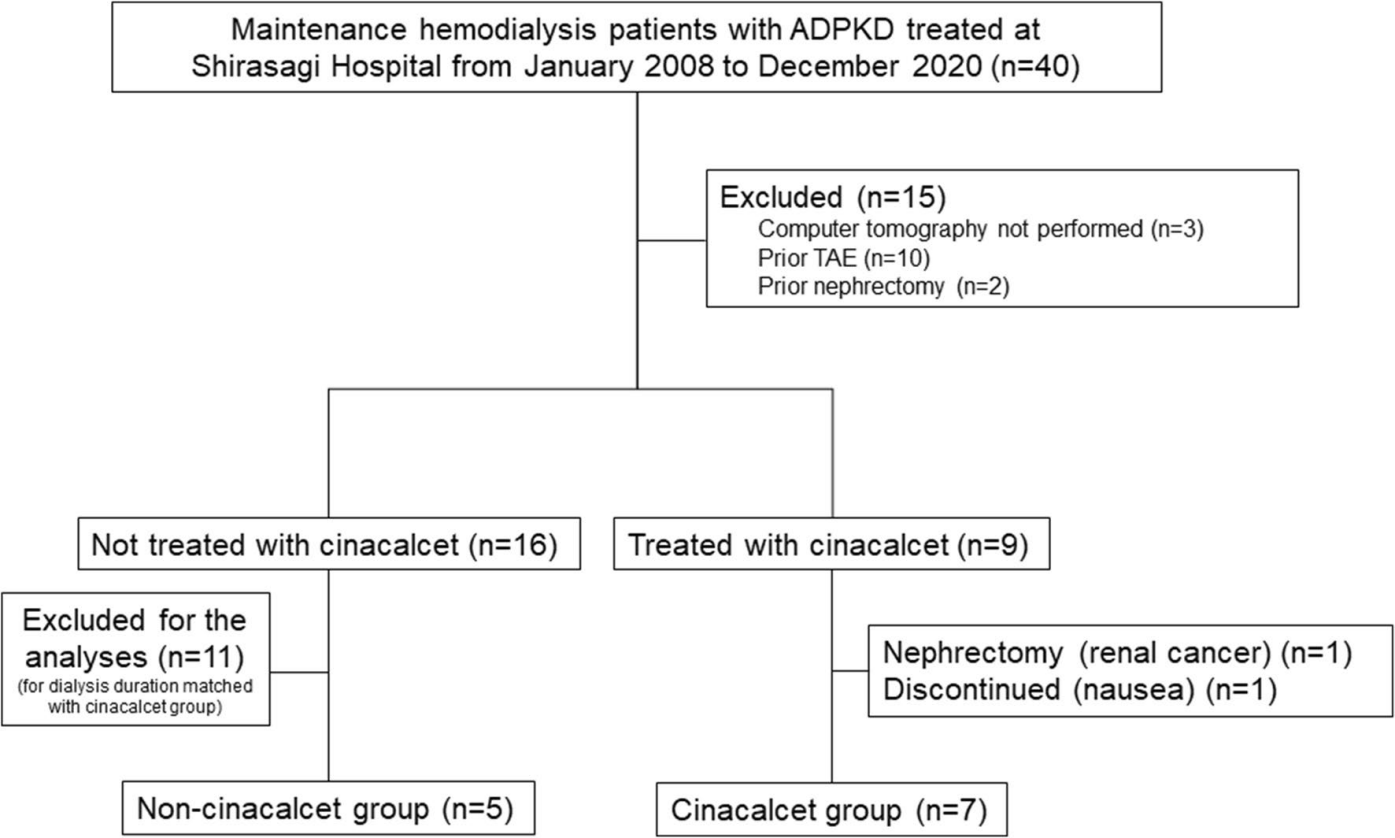
Flow of subject selection. This was a retrospective study of maintenance hemodialysis patients with ADPKD being treated at a single center, Shirasagi Hospital. Seven patients received and could continue cinacalcet treatment (cinacalcet group). Sixteen patients did not receive cinacalcet treatment. Of the 16, 5 patients (non-cinacalcet group) were chosen to match hemodialysis duration with the cinacalcet group.

A flow chart showing selection of the study participants is presented in Fig. 1. The clinical characteristics of the two groups of ADPKD patients are shown in Table 1. Hemodialysis duration for the cinacalcet group (n = 7) was 11.3 ± 5.7 years, while that for the non-cinacalcet group (n = 5) was 11.6 ± 1.7 years (*p* = 0.429) (Table 1). In the cinacalcet group, 4 (57.1%) were male and mean age at the initiation of treatment was 64.5 ± 9.8 years. Serum intact PTH, calcium, and phosphate levels were 489 ± 285 pg/mL, 9.6 ± 0.4 mg/dL, and 6.2 ± 1.3 mg/dL, respectively, indicating that all had secondary hyperparathyroidism according to the 2006 Japanese Guidelines for management of secondary hyperparathyroidism in dialysis patients^23^. In the non-cinacalcet group with longer hemodialysis duration, mean age at the first abdominal computed tomography (CT) examination after initiation of hemodialysis was 68.4 ± 8.6 years. Serum intact PTH was 195 ± 153 pg/mL, which was significantly higher in the cinacalcet group as compared to the non-cinacalcet group (*p* = 0.030).

**Table 1:** Baseline clinical characteristics of enrolled hemodialysis patients with autosomal dominant polycystic kidney disease (n = 12).

	Without cinacalcet^†^ (n = 5)	With cinacalcet^‡^ (n = 7)	*p *value
Gender (male/female)	4/1	4/3	0.836
Age (years)	68.4 ± 8.6	64.5 ± 9.8	0.845
Age at hemodialysis imitation (years)	57.2 ± 7.9	53.9 ± 9.4	0.604
Dialysis duration (years)	11.6 ± 1.7	11.3 ± 5.7	0.429
Total kidney volume (mL)	1,427 ± 386	2,644 ± 1508	0.147
Height-adjusted total kidney volume (mL/m)	881 ± 228	1,632 ± 919	0.106
Calcium (mg/dL)	9.0 ± 0.4	9.6 ± 0.4	0.069
Phosphate (mg/dL)	4.9 ± 1.3	6.2 ± 1.3	0.147
Intact PTH (pg/mL)	195 ± 153	489 ± 285	0.030
**Complications**			
Liver cyst N (%)	5 (100)	7 (100)	
Hypertension N (%)	3 (60)	7 (100)	
**Medications**			
Calcium carbonate, N (%)	3 (60)	5 (71.4)	
Sevelamer hydrochloride, N (%)	0 (0)	5 (71.4)	
Intravenous VDRA	1 (20)	4 (57.1)	
Oral VDRA	4 (80)	1 (14.3)	

Values are expressed as mean ± SD. Unpaired Student’s t-test was used for comparisons of continuous variables that exhibited a normal distribution. Mann–Whitney *U* test was used for comparisons of continuous variables with skewed distribution. Chi-squared test was used for comparisons of categorical variables.

*PTH* parathyroid hormone, *VDRA* vitamin D receptor activators.

^†^Obtained at the time of computed tomography examination.

^‡^Before starting cinacalcet treatment.

### Comparison of annual changes in TKV between cinacalcet and non-cinacalcet

**Figure 2:**
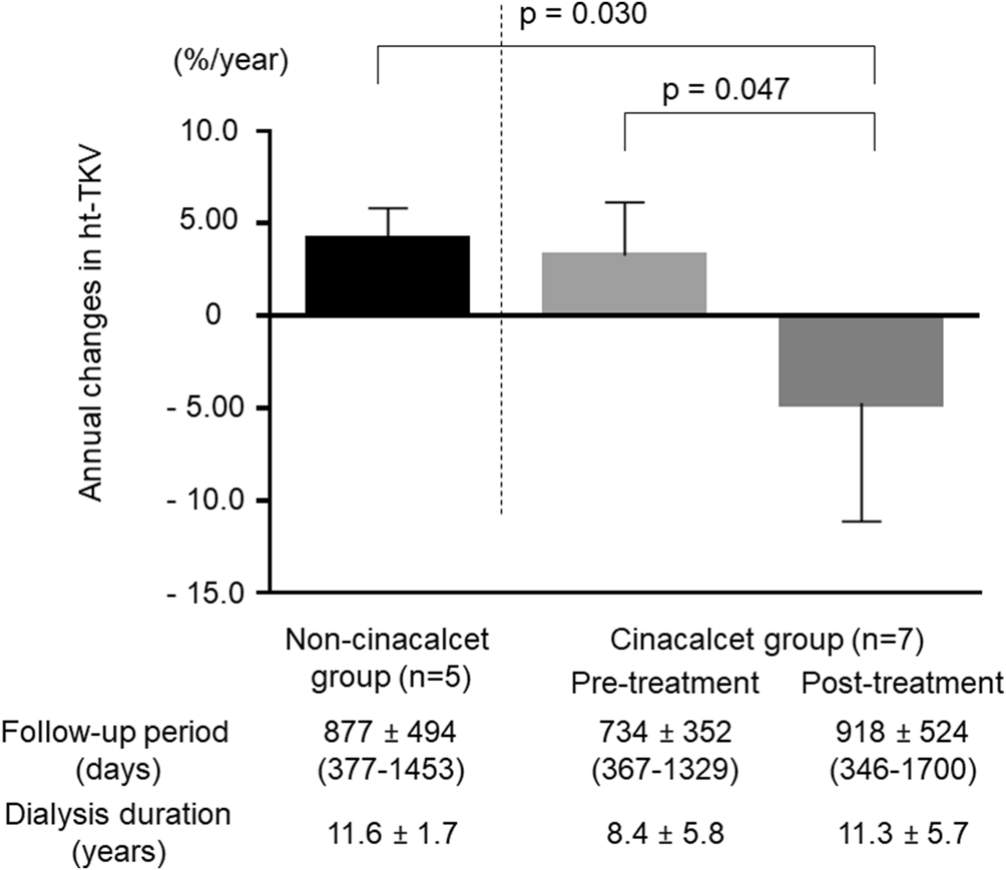
Annual changes in total kidney volume in hemodialysis patients with autosomal dominant polycystic kidney disease (ADPKD). Left: Non cinacalcet group. Center: Cinacalcet group before cinacalcet treatment. Right: Cinacalcet group after cinacalcet treatment. Cinacalcet treatment significantly suppressed kidney enlargements in ADPKD patients undergoing hemodialysis.

**Figure 3:**
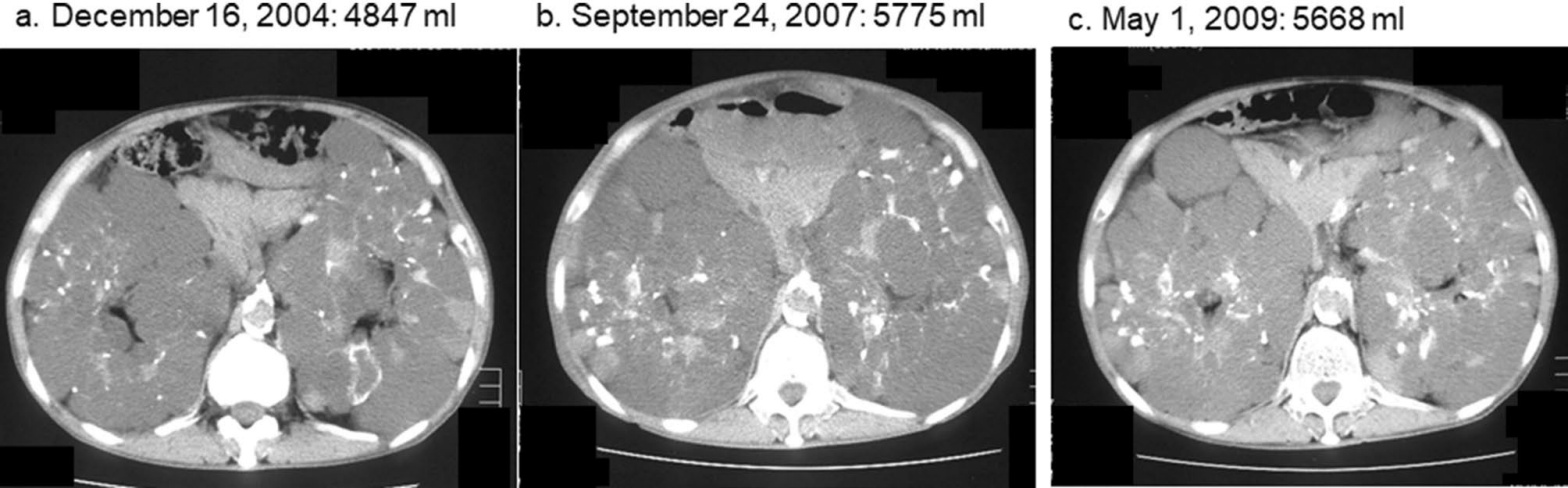
A representative case, abdominal computed tomography images (Case 3). Total kidney volume continued to increase after initiation of hemodialysis [(**a**) 4847 ml, December 16, 2004, (**b**) 5775 ml, September, 24 2007]. Cinacalcet administration was started on April 9, 2008. Total kidney volume did not increase during the cinacalcet treatment period [(**c**) 5668 ml, May 1, 2009].

Changes in TKV and height-adjusted TKV (htTKV) in the cinacalcet and non-cinacalcet groups are shown in Tables 2 and 3, respectively. Changes in htTKV in the cinacalcet and non-cinacalcet groups are also shown in Supplementary Figs. 1 and 2, respectively. In the non-cinacalcet group, the annual change in TKV rate was 4.19 ± 1.71% during a total period of 877 ± 494 days (range 377–1453 days) (Fig. 2), while that in the cinacalcet group before the start of cinacalcet treatment was 3.26 ± 2.87% during a period of 734 ± 352 days (range 367–1329 days), with the difference between the groups for those periods not significant (*p* = 0.755). However, following the start of cinacalcet treatment, the annual change in TKV rate in the cinacalcet group was − 4.71 ± 6.42% during a period of 918 ± 524 days (range 346–1700 days) (Fig. 2). During the treatment period, TKV increase was suppressed, with a representative case shown in Fig. 3. The changes of the log-transformed htTKV per month before and after the cinacalcet prescription were estimated as 0.00039 and -0.00312, respectively. Although the difference in the slopes was − 0.00351 (*p* = 0.214), the annual change in TKV rate was significantly decreased in the cinacalcet group after starting cinacalcet treatment as compared to that in the cinacalcet group before treatment started (*p* = 0.047). Although both the follow-up period and hemodialysis duration were not significantly different between the cinacalcet group after starting cinacalcet treatment and non-cinacalcet group, the annual change in TKV rate was significantly lower in the cinacalcet group after starting cinacalcet treatment as compared to that in the non-cinacalcet group (*p* = 0.030) (Fig. 2).

**Table 2:** Changes in total kidney volume (TKV) and height-adjusted total kidney volume (htTKV) in autosomal dominant polycystic kidney disease (ADPKD) patients of cinacalcet group (n = 7).

Patient no	TKV (mL) (htTKV (mL/m)) [hemodialysis duration (months)]											
1		1916	1926	1681	2056		2069	1835	1698	1730		
		(1147)	(1153)	(1007)	(1231)		(1239)	(1099)	(1017)	(1036)		
		[224]	[236]	[245]	[252]	[253]*	[258]	[263]	[275]	[289]		
2	2766	2811	2672	2756	2844		2390	1944	1683	1660		
	(1686)	(1714)	(1629)	(1681)	(1734)		(1457)	(1185)	(1026)	(1013)		
	[42]	[49]	[62]	[73]	[83]	[94]*	[95]	[99]	[125]	[138]		
3		4847	5697	6011	5775		5668	5503	5185			
		(2955)	(3474)	(3665)	(3521)		(3456)	(3355)	(3161)			
		[160]	[162]	[184]	[193]	[200]*	[213]	[219]	[225]			
4			3084	2937	3324		3652	3002	3189			
			(1848)	(1760)	(1991)		(2188)	(1798)	(1911)			
			[66]	[72]	[84]	[96]*	[96]	[105]	[107]			
5			1551	1621	1724		1681	1698	1662	1664	1770	1644
			(1055)	(1103)	(1173)		(1144)	(1155)	(1131)	(1131)	(1204)	(1119)
			[102]	[113]	[123]	[144]*	[146]	[151]	[162]	[177]	[189]	[202]
6				1267	1275		1093	983	1029	986		
				(831)	(836)		(717)	(645)	(675)	(647)		
				[39]	[51]	[67]*	[69]	[75]	[80]	[93]		
7				1856	1857		2058	2118	2302			
				(1105)	(1105)		(1225)	(1260)	(1370)			
				[73]	[85]	[91]*	[100]	[109]	[122]			

*ADPKD* autosomal dominant polycystic kidney disease, *TKV* total kidney volume, *htTKV* height adjusted total kidney volume.

*start of cinacalcet treatment.

**Table 3:** Changes in total kidney volume (TKV) and height-adjusted total kidney volume (htTKV) in autosomal dominant polycystic kidney disease (ADPKD) patients of non-cinacalcet group (n = 5).

Patient no	TKV (mL) (htTKV (mL/m)) [hemodialysis duration (months)]				
1	1114	1218	1244	1220	1321
	(688)	(752)	(768)	(753)	(815)
	[149]	[161]	[173]	[185]	[197]
2	1356	1487	1465		
	(812)	(890)	(877)		
	[111]	[126]	[138]		
3	2076	2149			
	(1274)	(1319)			
	[151]	[164]			
4	1427	1487	1723	1772	1776
	(862)	(898)	(1041)	(1071)	(1073)
	[122]	[134]	[140]	[152]	[165]
5	1160	1190			
	(772)	(792)			
	[176]	[189]			

*ADPKD* autosomal dominant polycystic kidney disease, *TKV* total kidney volume, *htTKV* height adjusted total kidney volume.

### Changes in intact PTH, calcium, and phosphate after cinacalcet treatment

Table 4 shows changes in serum calcium, phosphate, and intact PTH levels after treatment with cinacalcet in hemodialysis patients with ADPKD. During the cinacalcet treatment observational period, the average drug dosage was 28.7 ± 13.7 mg/day. Patients who received cinacalcet treatment showed significantly decreased serum intact PTH levels, from 489 ± 285 pg/mL at baseline to 229 ± 138 pg/mL at the end of the observation period (*p* = 0.032). Cinacalcet treatment also tended to cause a decrease in serum calcium and phosphate from the baseline to the end of the observation period [9.6 ± 0.4 to 9.3 ± 0.6 mg/dL (*p* = 0.191) and 6.2 ± 1.3 to 4.9 ± 0.9 mg/dL (*p* = 0.139), respectively]. Serum albumin, body mass index, and body weight were not significantly different between the baseline and final follow-up examination, suggesting that the nutritional status in those patients did not appreciably change during the follow-up period.

**Table 4:** Changes in serum calcium, phosphate, and intact-PTH levels, and total kidney volume before and after cinacalcet treatment in hemodialysis patients with autosomal dominant polycystic kidney disease, and cinacalcet amount (n = 7).

Patient no	Calcium (mg/dL)		Phosphate (mg/dL)		*Intact-PTH (pg/dL)		Average amount of cinacalcet (mg/day)
	Pre	Post	Pre	Post	Pre	Post	
1	9.9	9.7	4.5	5.9	546	81.4	24.3
2	9.4	9.1	8.5	4.1	543	490	50.8
3	9.6	10.3	5.4	5.8	1060	209	37.0
4	10.1	9.3	6.1	5.1	323	232	19.3
5	9.5	8.4	6.7	3.5	184	215	14.6
6	9.0	8.7	5.4	4.7	302	86	12.5
7	9.8	9.6	6.9	5.4	466	288	24.8
Mean ± SD	9.6 ± 0.4	9.3 ± 0.6	6.2 ± 1.3	4.9 ± 0.9	489 ± 285	229 ± 138	28.7 ± 13.7

*PTH* parathyroid hormone, *SD* standard deviation.

*p < 0.05.

## Discussion

The present study was conducted to investigate the effects of cinacalcet on progressive kidney enlargement in hemodialysis patients affected by ADPKD and with secondary hyperparathyroidism. Our findings demonstrated that cinacalcet treatment significantly suppressed an increase in TKV in the examined patients.

Previous studies have shown that TKV in ADPKD patients was decreased within 2–3 years after initiation of hemodialysis, likely due to a reduction in retained fluid secondary to a decrease in solute loading^24,25^. However, in maintenance hemodialysis patients with ADPKD with a longer hemodialysis duration, TKV usually continues to increase, because kidney blood flow is well preserved in the enlarged kidney arteries with preserved peripheral branches^4^. In the present study, TKV showed a tendency to gradually decrease in ADPKD patients within 3 years after initiation of hemodialysis, after which a gradual increase was seen (Supplementary Fig. 3). In addition, kidney volume changes in ADPKD patients undergoing hemodialysis and treated with cinacalcet were compared to those without cinacalcet treatment after adjusting for hemodialysis duration, since that duration may have effects on TKV increase.

Intracellular calcium and cAMP levels are regulated in the kidneys by extracellular CaSR activation^26,27^. CaSR has been identified in various organs, including expression in several different kidney tubular segments, such as the apical membrane of proximal tubules, basolateral membranes of the medullary thick ascending limb, cortical thick ascending limb, distal convoluted tubule, and apical surface of the inner medullary collecting duct^22,28,29^. Additionally, a recent in vitro study of human conditionally immortalized proximal tubular epithelial cells carrying the PKD1 mutation demonstrated that selective CaSR activation by NPS-R568 increased intracellular calcium and reduced cAMP levels^22^. In hemodialysis patients with secondary hyperparathyroidism, an allosteric modulator of CaSR treatment is clinically recommended, because that can decrease total parathyroid volume as well as the level of intact PTH in serum^30^. In the present study, the annual rate of increase in TKV before starting cinacalcet treatment was 3.26 ± 2.87%, which was significantly decreased to − 4.71 ± 6.42% after the cinacalcet treatment observation period. Although cAMP levels in serum and the kidneys were not evaluated in this retrospective investigation, the decreased cAMP level induced by cinacalcet treatment might be responsible for suppression of kidney enlargement in our patients.

Several in vivo studies have been conducted in which NPS-R568 was administrated in animal models of PKD. NPS-R568 administration in *pcy* mice, a nephronophthisis (NPHP) orthologous animal model, inhibited cyst growth and renal fibrosis progression, and significantly reduced kidney weights in the later stage of NPHP^31^. In another study, NPS-R568 was administrated to heterozygote Cy/+ rats, an animal model of ADPKD, from 20 to 38 weeks of age, and kidney and cyst volumes were shown to be significantly lower as compared to rats without NPS-R568 treatment that received calcium^32^. In contrast, a study that used PCK rats, a model of autosomal recessive polycystic kidney disease, from 10 to 16 weeks of age, and also *Pkd2*^−*/WS25*^ mice, a mild phenotype of ADPKD, found no detectable effects of NPS-R568 on cystogenesis^33^. The discrepancy of reported effects of NPS-568 may be possibly due to different stages of PKD and kidney function. In the study that used PCK rats and *Pkd2*^−*/WS2*5^ mice, the animals were younger and the stages of kidney dysfunction were milder. In contrast, the animals used in experiments of NPHP mice and Cy/+ rats were older, and also showed enlarged kidneys and advanced kidney failure. Notably, administration of NPS-R568 to Cy/+ rats aged from 20 to 34 weeks did not change kidney or cyst volumes, as compared to those without treatment or with calcium treatment used as a placebo^32^. Those previous results along with the present findings suggest that the effects of CaSR agonist might be limited to later or advanced stages of ADPKD, particularly in affected patients with kidney failure. The efficacy of tolvaptan to prevent enlargement of the kidneys and kidney function deterioration in pre-dialysis ADPKD patients has been established^17,18^. In contrast, tolvaptan is contraindicated for ADPKD patients with kidney failure. For CKD stage 1–4 ADPKD patients, following treatment with tolvaptan, administration of cinacalcet for CKD stage 5D might be a rational strategy to suppress kidney enlargement.

PTH is positively coupled to generation of cAMP via the action of PTH receptor 1 (PTH1R)^34^. One possible mechanism by which cinacalcet suppressed kidney enlargement in the present cohort is by lowering the level of PTH in serum, which leads to reduced tubular activation of PTH1R and cAMP production in kidney cyst cells. Since PTH increases both serum and kidney cAMP levels, we examined the relationship between change in PTH level and TKV in the present ADPKD patients. However, even though the cinacalcet group showed significantly higher serum PTH levels as compared to the non-cinacalcet group, the annual rate of TKV increase was similar between them before cinacalcet treatment. Six of the 16 patients in the non-cinacalcet group were treated with intravenous VDRA and TKV was increased at a similar rate. Therefore, it is considered that cinacalcet has a beneficial effect to suppress kidney enlargement, independent of the level of PTH in serum.

Intervention to reduce TKV can cause complications^4^. Although TAE is an effective procedure, complications such as severe pain within 5 days and fever greater than 38 °C persisting for up to eight days can occur within the first week after treatment^4^. As compared to interventions to reduce TKV, CaSR agonist are safer, though one of the present patients discontinued cinacalcet due to nausea. A recent meta-analysis of related trials reported risk ratios related to cinacalcet treatment as compared to a placebo in hemodialysis patients of 8.37 for hypocalcemia, 2.10 for nausea, and 2.01 for vomiting^35^. Although hypocalcemia is the main adverse event associated with cinacalcet treatment, it is relatively easily controlled with use of a VDRA and/or calcium-containing phosphate binders.

The present study has some limitations. First, the number of hemodialysis patients examined was relatively small, mainly due to the fact that the enrolled study subjects were treated at a single institution and the prevalence of ADPKD in patients undergoing hemodialysis is only 2–3% in Japan. Furthermore, since patients with secondary hyperparathyroidism who received cinacalcet treatment and underwent abdominal CT examinations were needed, it was difficult to obtain an adequate number. Second, all of the patients were of Japanese ethnicity. In the TEMPO 3:4 trial, the efficacy of tolvaptan for TKV was more effective in Japanese, in whom the annual TKV increase rate was 1.3%, as compared to subjects with a different ethnicity, in whom the annual increase was 2.8%^36^. Therefore, the results obtained from this study conducted in Japan might not be applicable to other ethnicities. Third, this was performed as a retrospective observational study, possibly limiting conclusions to be made from the findings. Fourth, the method used for measuring TKV in the present study was simple^37^, though more precise qualitative assessment of TKV is available. Furthermore, the CT findings obtained could not be analyzed with newer advanced systems, as those were not available. A fifth limitation is that total liver volume could not be evaluated. In some of the present cases, there were no apparent changes after cinacalcet treatment (Supplementary Fig. 4), findings consistent with those in a previous in vivo study^33^. In contrast, CaSR is also expressed in hepatocytes^38,39^ and a recent meta-analysis revealed that somatostatin analogue treatment improved total liver volume in ADPKD patients^40^. Thus, cinacalcet may also have effects on liver cysts, though additional studies are needed to assess such effects in ADPKD patients. Another limitation is that though none of the present patients were treated with tolvaptan or somatostatin analogue, they may have taken other unknown medications that might have influenced the TKV increase seen in ADPKD patients undergoing hemodialysis. Seventh, other novel calcimimetic agents, such as etelcalcetide or evocalcet, were not evaluated in the present study. Finally, the clinical outcomes of enlarged kidney-related complications including gastrointestinal symptoms or quality of life could not be evaluated. The efficacy of treatments performed for these symptoms as well as quality of life are relevant factors. A previous study of 188 ADPKD patients undergoing hemodialysis demonstrated that kidney TAE was effective for improving abdominal fullness, appetite, and 36-item Short Form healthy Survey (SF-36) scores, such as the mental component and role/social component summaries^41^. Whether cinacalcet has a beneficial effect on those outcomes should be investigated in future study. Lastly, in the cinacalcet group, although the annual rate of increase in htTKV before cinacalcet treatment was significantly suppressed after cinacalcet treatment, the difference in the slopes of htTKV changes between the periods before and after cinacalcet treatment did not reach a statistically significance. It may be because the timing of CTs, duration of cinacalcet treatment, and htTVK in each patient were very varied, and also because the number of patients was small. Future well-designed prospective longitudinal or randomized control studies are needed to investigate whether various calcimimetic agents are able to suppress kidney enlargement as well as enlarged kidney-related symptoms for improving quality of life in hemodialysis patients with ADPKD that use more precise evaluation methods of outcomes.

The present findings are the first to demonstrate that cinacalcet suppresses kidney enlargement in hemodialysis ADPKD patients with secondary hyperparathyroidism. Cinacalcet treatment may be helpful for patients with ADPKD who are undergoing hemodialysis to suppress progressive enlargement of the kidneys and also relieve enlarged kidney-related clinical problems including gastrointestinal symptoms. Additionally, that may also postpone the requirement of a kidney volume reduction procedure, such as nephrectomy or TAE, for ADPKD patients requiring hemodialysis. Thus, we propose that cinacalcet treatment be considered as an initial therapeutic option to control secondary hyperparathyroidism in ADPKD hemodialysis patients rather than a VDRA. Furthermore, cinacalcet may possibly suppress kidney enlargement even in patients without secondary hyperparathyroidism, though additional large scale studies are needed. In summary, cinacalcet showed protective effects when given to ADPDK patients, and potentially suppresses kidney enlargement as well as hyperparathyroidism.

## Methods

### Ethics statement

This study was performed according to the ethical guidelines for medical and health research involving human subjects by the Japanese Ministry of Health, Labour and Welfare, and the Declaration of Helsinki. The protocol of this investigation was approved by the Ethics Committee of Shirasagi Hospital (No. 2019005). Written informed consent prior to the study was obtained from the subjects or an opt-out option was chosen, as explained in instructions posted on the website of the institution.

### Patients

Thi s was a retrospective observational study. Initially, we evaluated 40 maintenance hemodialy4.2sis patients with ADPKD who underwent 3- to 4-h hemodialysis sessions 3 times a week at Shirasagi Hospital from January 2008 to December 2020. Changes in TKV were not evaluated in 15, as CT imaging results were not adequate (n = 3), or TAE (n = 10), or as nephrectomy (n = 2) had been already performed at the time of the screening. Of the 25 patients whose TKV changes were evaluated, 9 were scheduled to be treated with cinacalcet hydrochloride. Of whom 1 could not continue cinacalcet due to nausea and a nephrectomy was performed in 1 for kidney cancer. Finally, 7 patients were included in the cinacalcet group. Of the 16 patients not treated with any CaSR, 5 with dialysis duration matched with that of the cinacalcet group were evaluated as controls (non-cinacalcet group), since dialysis duration affected TKV volume (Fig. 1).

### Blood sampling

Blood samples were obtained from the arteriovenous fistula just prior to the first hemodialysis session of the week, and routine laboratory tests were performed within 3 h of blood sampling using an automated analyzer^42,43^. Serum parameters, including intact PTH, calcium, and phosphate, were measured close to the time of the CT examinations, and used for statistical analyses. Serum calcium was corrected by the value for serum albumin as follows. When serum albumin was < 4.0 g/dL, then the following formula was used: corrected calcium (cCa) (mg/dL) = measured Ca (mg/dL) + [4 − measured albumin (g/dL)]^23^.

### Treatment

The main entry criteria of cinacalcet treatments were baseline serum levels of intact PTH and calcium greater than 180 pg/mL and 9.0 mg/dL, respectively, even after treatment with VDRAs and/or phosphate binders. Cinacalcet was started at a daily dose of 25 mg, and then gradually adjusted from 12.5 to 75 mg/day when necessary to achieve a target serum intact PTH level between 60 and 180 pg/mL, as defined by the 2006 Japanese Guidelines for management of secondary hyperparathyroidism in chronic dialysis patients^23^.

### Computed tomography imaging for total kidney volume (TKV)

The kidneys in the present patients were evaluated using CT examinations. At Shirasagi hospital, abdominal CT scans were basically taken almost every year to medical check-up for hemodialysis patients. TKV determination using CT results was performed at least twice, both before and after cinacalcet treatment, with linear kidney dimensions (length, lateral diameter, anterior–posterior diameter) measured by K.N., who was blinded to the clinical data. The lengths of both kidneys were calculated from axial slices by multiplying slice thickness by number of slices between the superior and inferior tips of the kidneys, as previously described^37^. TKV was estimated from the linear dimensions using an ellipsoid formula, as follows: length × width × thickness × π/6^44–46^.

Annual changes were calculated as follows.

Non-cinacalcet group:

$$ {\text{Annual change in TKV }}(\% {\text{/year}}) = [({\text{TKV at most recent CT)}} - ({\text{TKV at oldest CT)}}]{/}({\text{TKV at oldest CT}}){/}({\text{days between CT examinations/365days}}) \times {100} $$

Cinacalcet group before cinacalcet treatment:

$$ {\text{Annual change in TKV }}(\% {\text{/year}}) = [({\text{TKV at most recent CT before starting cinacalcet)}} - ({\text{TKV at oldest CT)}}]{/}({\text{TKV at oldest CT}}){/}({\text{days between CT examinations/365 days}}) \times {100} $$

Cinacalcet group after cinacalcet treatment:

$$ {\text{Annual change in TKV }}(\% {\text{/year}}) = [({\text{TKV at most recent CT after starting cinacalcet)}} - ({\text{TKV at next CT after starting cinacalcet)}}]{/}({\text{TKV at next CT after starting cinacalcet}}){/}({\text{days between CT examinations/365 days}}) \times {100} $$

### Statistical analysis

Continuous variables are expressed as the mean ± SD, and categorical variables as numbers and percentages. Student’s t-test or Mann–Whitney *U* was used for comparisons of clinical parameters between ADPKD patients with and without cinacalcet treatment. Paired Student’s t-tests or Wilcoxon signed-rank test were used for comparisons of clinical parameters of ADPKD patients who received cinacalcet between pre-administration and follow-up examinations. Correlation and linear regression analyses were done to examine relationships between TKV change and clinical parameters. In the cinacalcet group, the change of htTKV over time was assessed using a mixed-effect model with a random intercept for each patient. In this model, the difference in the trends between the periods before and after the cinacalcet prescription was also evaluated. To satisfy a normality assumption of an error term, the outcome variable was used with natural log-transformation. Statistical analyses were performed using GraphPad Prism, version 6.0 (GraphPad Software, San Diego, CA, USA) or the JMP software package, version 10 (SAS Institute, Inc., Cary, NC, USA), or R version 4.0.3 (https://cran.r-project.org/). *p* values < 0.05 were considered to indicate statistical significance.

## Supplementary Information

Supplementary Information 1.

Supplementary Information 2.

Supplementary Information 3.

Supplementary Information 4.

Supplementary Information 5.

## Abbreviations

ADPKD: Autosomal dominant polycystic kidney disease
cAMP: Adenosine-3′,5′-cyclic monophosphate
CaSR: Calcium sensing receptor
CKD: Chronic kidney disease
CT: Computed tomography
htTKV: Height adjusted total kidney volume
KRT: Kidney replacement therapy
NPHP: Nephronophthisis
PTH: Parathyroid hormone
PTH1R: PTH receptor 1
TKV: Total kidney volume
TAE: Transcatheter arterial embolization
VDRA: Vitamin D receptor agonists
