# Supplementary material for: Cinacalcet may suppress kidney enlargement in hemodialysis patients with autosomal dominant polycystic kidney disease
Source: Sci Rep. 2021 May 11;11:10014. doi: 10.1038/s41598-021-89480-1 (PMC8113347; doi:10.1038/s41598-021-89480-1)
Supplement: Supplementary file 1 — Supplementary Information 1. [file 41598_2021_89480_MOESM1_ESM.docx]

Supplementary Figure 1. Changes in height adjusted total kidney volume (htTKV) in cinacalcet group.

In hemodialysis patients with autosomal dominant polycystic kidney disease (n=7), increase of htTKV was suppressed after initiation of cinacalcet. Arrow indicates the initiation of cinacalcet treatment.

Supplementary Figure 2. Changes in height adjusted total kidney volume (htTKV) in non-cinacalcet group.

In autosomal dominant polycystic kidney disease with longer hemodialysis duration (n=5), htTKV continues to increase.

Supplementary Figure 3. Sort term changes in height adjusted total kidney volume (htTKV) after initiation of hemodialysis in 11cases.

htTKV tended to decrease during 2-3 years after initiation of hemodialysis. After then, it gradually tended to increases in autosomal dominant polycystic kidney disease patient without cinacalcet treatment (n=11).

Supplementary Figure 4. A representative case, abdominal computed tomography images (Case 6).

Liver cysts were not changed before (a: November, 25 2015) and after cinacalcet treatment period (b: May, 15 2019)
