# Supplementary figures and images for: Cinacalcet may suppress kidney enlargement in hemodialysis patients with autosomal dominant polycystic kidney disease
Source: Sci Rep. 2021 May 11;11:10014. doi: 10.1038/s41598-021-89480-1 (PMC8113347; doi:10.1038/s41598-021-89480-1)

Supplementary Figure 1

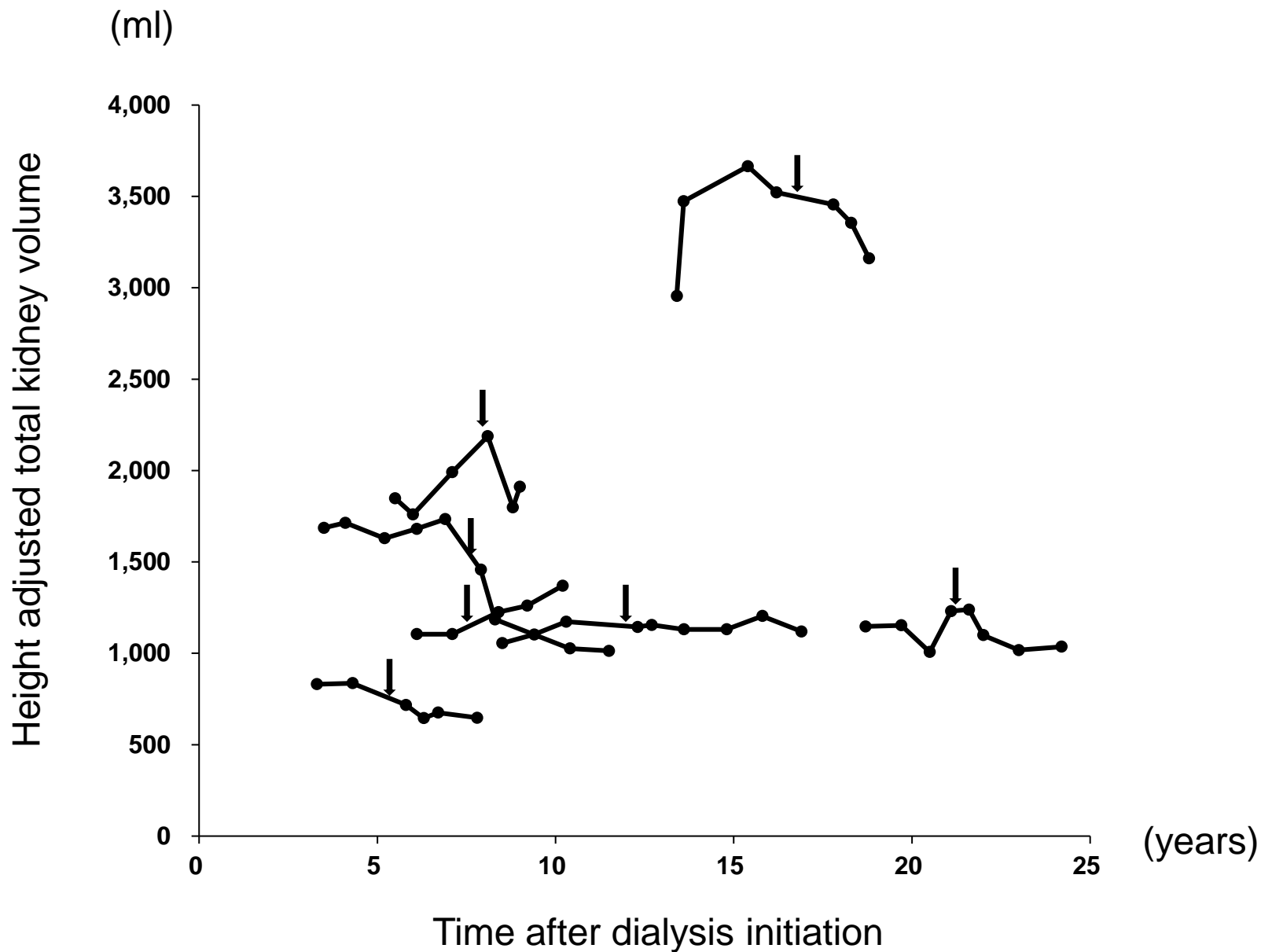

Supplement: Supplementary file 2 — Supplementary Information 2. [file 41598_2021_89480_MOESM2_ESM.pdf]

Supplementary Figure 2

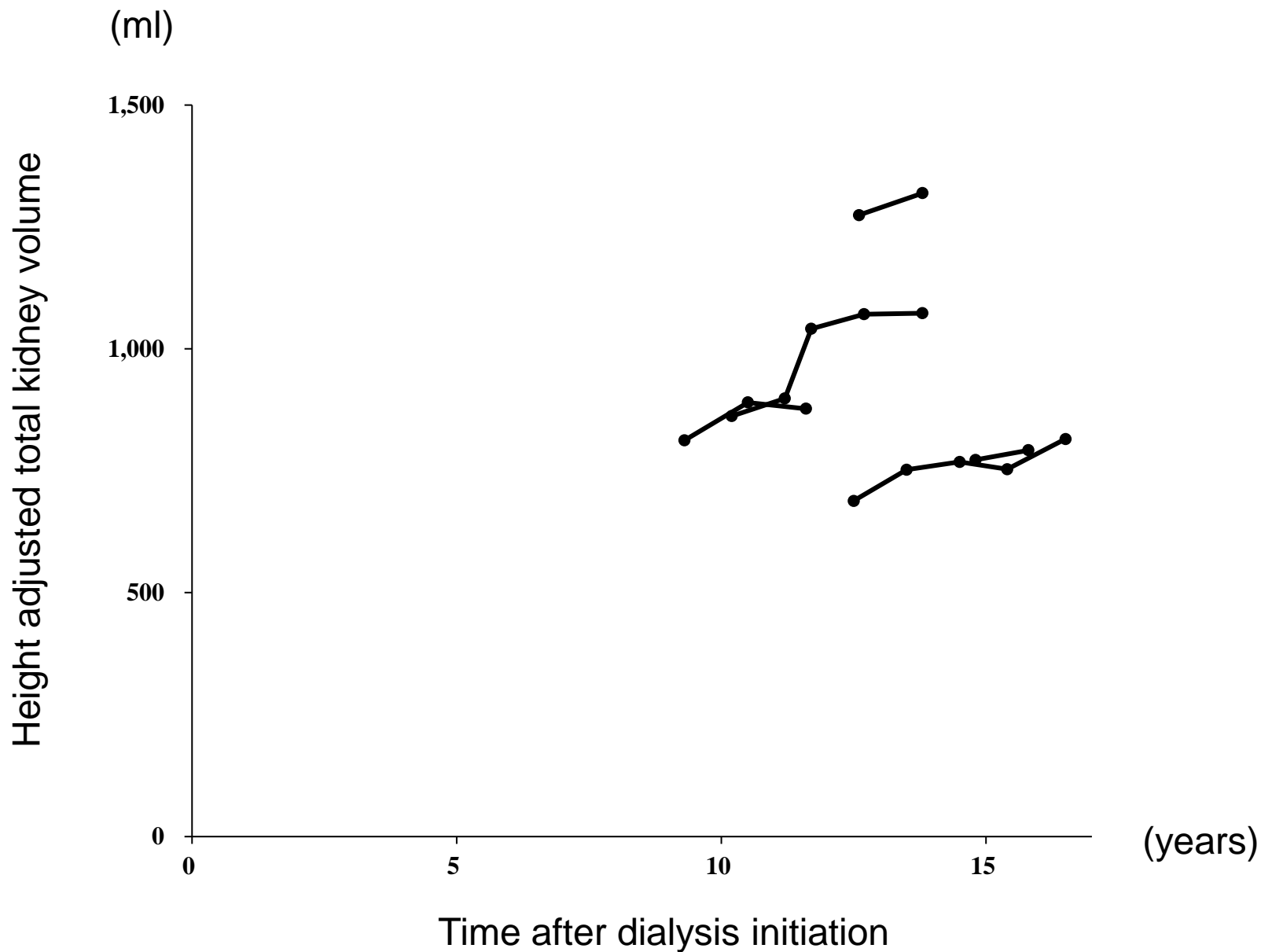

Supplement: Supplementary file 3 — Supplementary Information 3. [file 41598_2021_89480_MOESM3_ESM.pdf]

Supplementary Figure 3

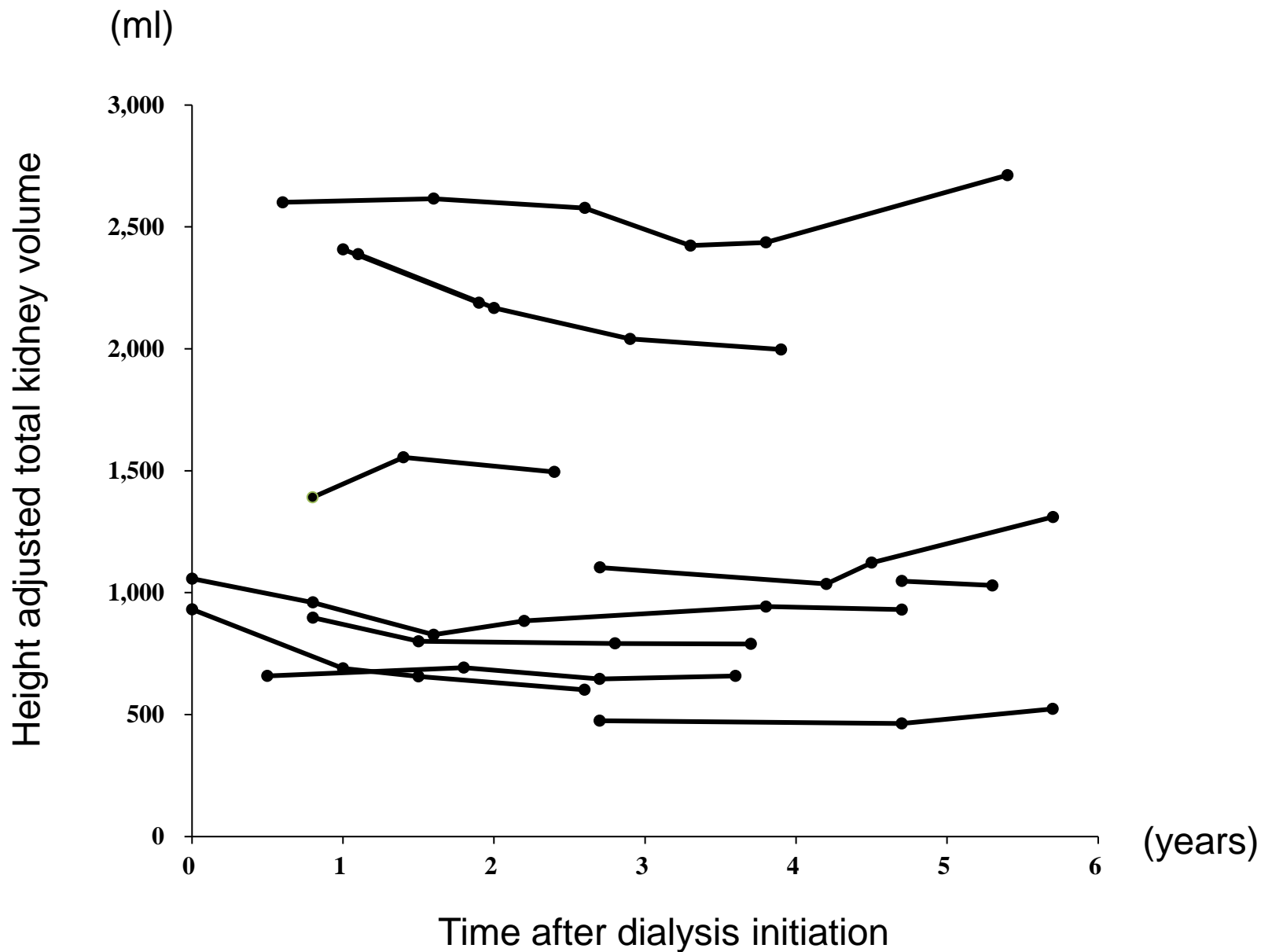

Supplement: Supplementary file 4 — Supplementary Information 4. [file 41598_2021_89480_MOESM4_ESM.pdf]

a. November 25, 2015  
(16 month before cinacalcet treatment)

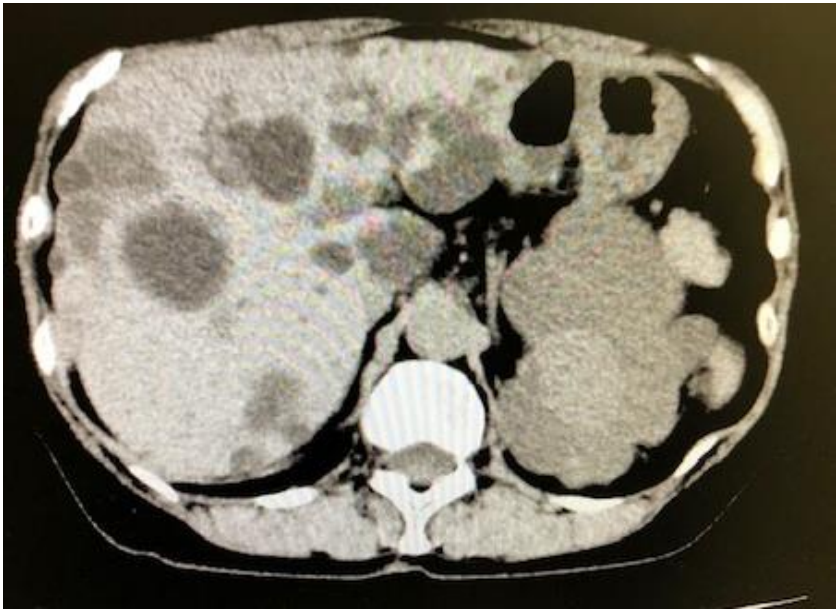

b. May 15, 2019  
(26 month after cinacalcet treatment)

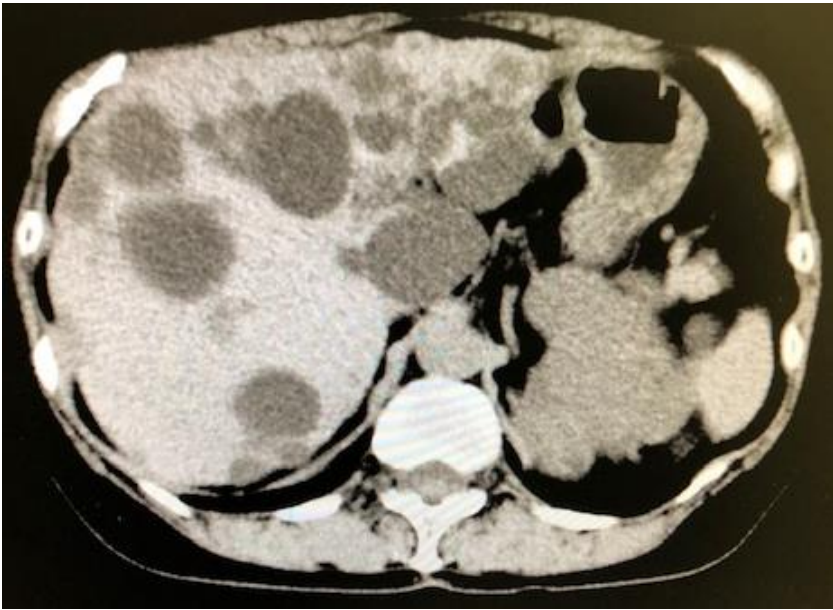

Supplement: Supplementary file 5 — Supplementary Information 5. [file 41598_2021_89480_MOESM5_ESM.pdf]
